# Analysis of ICU Nurses’ Needs for Intelligent Excreta Nursing Robots: A Multicenter Cross-Sectional Study in China

**DOI:** 10.3390/healthcare14152386

**Published:** 2026-08-04

**Authors:** Jingyu Li, Shuang Liang, Jinghao Chen, Fang Wang, Na Zhang, Yan Wang, Lu Chen

**Affiliations:** 1 School of Nursing, Nanjing University of Chinese Medicine, Nanjing 210029, China; lijingyu7744@163.com (J.L.); 20231024@njucm.edu.cn (S.L.); ojinghaoo@163.com (J.C.); 2 Department of Nursing Research Institute, Nanjing University, Nanjing 210008, China; wfxhch@163.com (F.W.); 230209221@seu.edu.cn (N.Z.); 3 School of Nursing, Dalian Medical University, Dalian 116044, China; 2224105400033@dmu.edu.cn

**Keywords:** critical care, excretion management, robotics, Kano, AHP

## Abstract

**Highlights:**

**What are the main findings?**

- Based on the Kano model, the 16 secondary requirement items for the intelligent excreta nursing robot were classified into 5 must-be, 3 one-dimensional, 6 attractive, and 2 indifferent attributes. Requirements with different attributes influence user satisfaction through distinct pathways.
- Based on the Analytic Hierarchy Process (AHP) calculations, assuming a total overall importance of 100%, the importance weights of the first-level requirement items were ranked in descending order as follows: safety assurance (36.89%), high-quality excreta handling function (30.29%), sustainable usage (22.21%), and harmonious human–machine–environment interaction (10.61%).

**What are the implications of the main findings?**

- With “safety assurance” as the core principle, anomaly prevention and emergency response, minimization of use-related injury risks, and comprehensive privacy protection are established as the absolute prerequisites for the robot’s design. Furthermore, significant emphasis is placed on refining the core technologies underlying the “high-quality excreta handling function,” specifically precise excrement sensing and the efficient cleansing of urine and feces.
- To ensure the sustainable utilization of robots, it is imperative to focus not only on the conventional requirements for a durable and robust design but also on guaranteeing reliable after-sales support. Furthermore, regarding robotic interactions, emphasis must be placed on fostering harmonious engagement among the robot, patients, and nursing staff.

**Abstract:**

**Background/Objectives**: As the number of critically ill patients continues to rise, intensive care unit (ICU) nurses bear a heavy nursing workload, within which excretion care is particularly time-consuming and labor-intensive. Although intelligent excreta nursing robots hold the potential to alleviate this burden and improve the quality of care, their widespread application in the ICU setting remains limited. Therefore, systematically analyzing the specific needs of ICU nurses is of profound significance for guiding the research and development of clinically adaptable robotic systems. **Methods**: A questionnaire survey was conducted in this study. Kano model-based questionnaires were administered to 401 clinical intensive care unit (ICU) nurses from 83 hospitals in China to analyze requirement attributes, and AHP-based questionnaires were distributed to 102 experts to evaluate the importance of these requirements. **Results**: Among the 16 s-level need items identified via the Kano model, five were classified as must-be quality attributes, where non-fulfillment significantly reduced satisfaction with the robot; three as one-dimensional quality attributes, demonstrating a positive linear correlation between fulfillment and user satisfaction; six as attractive quality attributes, where fulfillment substantially enhanced satisfaction; and two as indifferent quality attributes, exerting minimal impact on satisfaction. Furthermore, the AHP determined the relative importance weights of the first-level need items (totaling 100%), ranked in descending order as follows: safety assurance (36.89%), high-quality excreta handling function (30.29%), sustainable usage (22.21%), and harmonious human–machine–environment interaction (10.61%). **Conclusions**: In conclusion, the priority sequence for fulfilling ICU nurses’ requirements regarding intelligent excreta nursing robots dictates that safety assurance must serve as the fundamental prerequisite. This is followed by refining the core technologies for high-quality excreta handling, addressing key demands for sustainable usage and harmonious human–robot interaction, and ultimately accommodating other secondary needs.

## 1. Introduction

With the accelerating aging of the population, the number of patients admitted to intensive care units (ICUs) continues to rise [1]. The ICU is widely recognized as the clinical setting with the highest nursing intensity within a hospital. Here, critically ill patients undergo numerous complex procedures and are typically completely reliant on nursing staff for their basic daily care needs [2]. Notably, nurses perform excretion care approximately six times per day on average, representing a substantial proportion of their overall clinical workload. This high frequency is imperative, as inadequate excretion management can precipitate severe complications, such as pressure injuries [3]. Consequently, excretion care is universally regarded as one of the most arduous and physically demanding nursing tasks, highlighting an urgent need for strategies to alleviate the workload associated with this care [4].

In recent years, nursing robots have been increasingly explored in clinical settings. The intelligent excreta nursing robot is a type of device designed to partially or fully assist in the management and clearance of human excreta (urine and feces) [3]. Current research on intelligent excreta nursing robots spans both engineering and nursing disciplines; however, their primary foci differ considerably. Engineering research primarily concentrates on the technological development and performance optimization of these robots [5,6], such as mechanical structure design and motion control. Conversely, nursing research predominantly investigates the impact of these robots on nurses’ workload and the overall quality of patient care [7,8]. Studies suggest that the successful clinical implementation of robots depends not only on their technical performance but is also significantly influenced by user experience, organizational culture, workflow integration, and contextual adaptability [9,10]. However, existing research on intelligent excreta nursing robots is largely confined to home and nursing home settings, leaving a critical gap regarding high-risk, specialized environments like the intensive care unit (ICU); consequently, the intelligent excreta nursing robot has not yet been widely adopted in ICUs [8]. A synthesis of current literature reveals that a lack of systematic analysis grounded in clinical needs impedes the wider implementation of existing robots in the ICU. For instance, during the research and development phase of some robots, a profound understanding of nursing workflows and patient safety risks is often absent. This creates a discrepancy between robotic design and nurses’ expectations. Nurses expect devices that are compatible with concurrent clinical operations and equipped with robust safety safeguards. Consequently, this misalignment hinders the clinical translation of these technologies [11]. Furthermore, previous studies have predominantly utilized singular tools to evaluate satisfaction, acceptance, or usability, rarely incorporating both demand attributes and demand importance to systematically analyze user requirements [12]. Consequently, accurately determining the priority ranking of these needs remains challenging. Core frameworks in implementation science, such as the Consolidated Framework for Implementation Research (CFIR), deconstruct the clinical implementation process of medical technologies into five domains: intervention characteristics, outer setting, inner setting, characteristics of individuals involved, and the process of implementation [13]. Furthermore, CFIR explicitly indicates that the mismatch between technology and the specific clinical context is a systemic root cause of implementation failure [14]. The Non-adoption, Abandonment, Scale-up, Spread, and Sustainability (NASSS) framework further reveals that the structural misalignment between technological complexity and users’ value perceptions is a core mechanism hindering the broader adoption of medical technologies [15]. Consequently, a proactive and systematic needs analysis serves as a critical pathway to bridge the gap between technological design and clinical implementation, rather than merely being an appended step post-development. Furthermore, the Technology Acceptance Model (TAM) indicates that nurses’ perceived usefulness and perceived ease of use regarding robots are the primary determinants of their intention for continued use [16]. In the intensive care unit (ICU), where patients are frequently sedated, intubated, or experiencing altered levels of consciousness, nurses act as both the primary direct users and proxy assessors for patients. Therefore, their perceived needs and propensity for technology acceptance directly influence the successful implementation and promotion of robotic systems. Therefore, systematically analyzing nurses’ needs regarding the intelligent excreta nursing robot has emerged as a critical imperative to advance these devices from technical feasibility to successful clinical translation.

Guided by the Attractive Quality Theory, this study integrates the Kano model with the Analytic Hierarchy Process (AHP) to conduct a needs analysis of ICU nurses regarding intelligent excreta nursing robots. This approach aims to provide a user-centered reference for the research, development, and clinical implementation of such robots. The Attractive Quality Theory evaluates service attractiveness based on customer perception and the fulfillment of quality attributes, with a strong emphasis on user satisfaction. According to this theory, the presence or absence of a service attribute does not universally dictate customer satisfaction; rather, a nonlinear correlation exists between the fulfillment of specific service elements and overall user satisfaction [17]. As a methodological tool derived from this theory, the Kano model categorizes user needs into five distinct attributes based on user responses to varying degrees of requirement fulfillment, thereby elucidating how individual needs influence user satisfaction [18]. While the traditional Kano model primarily focuses on classifying requirement attributes, it falls short in quantifying the relative importance of individual needs. Therefore, this study further incorporates the AHP. By constructing a judgment matrix and conducting pairwise comparisons, the AHP translates complex, multi-level requirements into quantifiable weights. These weights are subsequently adjusted based on the Kano attributes to precisely determine the relative importance of each need [19]. The integration of the Kano model and AHP not only effectively delineates the pathways through which the fulfillment of individual need items impacts user satisfaction, but also quantitatively evaluates the relative importance of each item within the comprehensive needs framework. Ultimately, this combined methodology provides a robust scientific basis for the research, development, and functional optimization of intelligent nursing equipment and related products. This research design is highly consistent with the principles of Human-Centred Design (HCD). HCD requires the identification of user needs in the early phases of product development and the subsequent establishment of design priorities based on these needs [20].

Against this backdrop, this study systematically analyzed the multidimensional needs of ICU nurses regarding intelligent excreta nursing robots. To achieve this, we employed a cross-sectional survey approach combining the Kano model and AHP. This design was theoretically guided by the integration of the CFIR framework, HCD principles, and TAM. This study aims to elucidate the differential impact pathways of various need items on user satisfaction, quantify the relative importance of each item, and establish a sequence of design priorities. Ultimately, the goal is to provide a reference framework with both theoretical foundations and practical guidance for the functional configuration and clinical implementation of intelligent excreta nursing robots in ICU settings.

## 2. Materials and Methods

### 2.1. Study Design

A cross-sectional survey study design was applied.

### 2.2. Participants

A multi-stage sampling method was employed in this study. Based on the China Health Statistical Yearbook [21], China was divided into three regions: eastern, central, and western. More than five provinces, autonomous regions, or municipalities were randomly selected from each region. Subsequently, at least one hospital was selected from each sampled province, autonomous region, or municipality. Within these selected hospitals, the requirement attribute analysis questionnaire was distributed to nurses who met the inclusion and exclusion criteria, while the requirement importance analysis questionnaire was distributed to nurses who met the criteria for experienced nursing experts. The requirement attribute analysis questionnaire aims to identify how each requirement item impacts user satisfaction. It emphasizes subjective feelings and reflects the perception of needs in actual clinical scenarios. Therefore, the inclusion criteria for nurses completing this questionnaire were as follows: (1) working experience in the ICU ≥ 1 year; (2) direct involvement in or management of patients’ excretion care in routine work; (3) ability to correctly understand the questionnaire and cooperate well with the researchers; and (4) voluntary participation in this study. The exclusion criterion was: (1) an actual clinical working time of <6 months within the past year due to various reasons (e.g., sick leave, maternity leave, or study). The requirement importance analysis questionnaire conducts a pairwise comparison of the importance of each requirement item. This judgment process involves systematic trade-offs and holistic decision-making across multiple items. Therefore, it requires experienced nursing experts. These experts utilize a macroscopic cognitive framework, formed through long-term clinical practice or nursing management, to make more forward-looking and rational judgments. Therefore, the AHP-based requirement importance analysis questionnaire was completed by experienced nursing experts. The inclusion criteria for experienced nursing experts were: (1) working experience in the ICU ≥ 10 years; (2) relevant research experience or active interest in the content of this study; (3) an intermediate professional title or above; and (4) voluntary participation and cooperation in completing this study. According to Kendall’s sample size estimation method, the sample size for a cross-sectional study should be 5–10 times the number of independent variables [22]. For the Kano model-based requirement attribute analysis questionnaire, the sample size was calculated as 5–10 times the 32 questions (consisting of one positive and one negative question for each of the 16 items in the main section of the questionnaire). Factoring in an estimated 20% invalid response rate, a minimum of 200 participants was required to complete this questionnaire. Furthermore, large-scale simulation tests have confirmed that the sample size for AHP-based questionnaire surveys should be greater than 19 [23]. Accounting for a 20% invalid response rate, the AHP-based requirement importance analysis questionnaire required a minimum of 24 experienced nursing experts. Ultimately, this study included 401 ICU nurses for the Kano survey and 102 experienced nursing experts for the AHP survey, recruited from a total of 83 hospitals across 14 provinces, 2 autonomous regions, and 4 municipalities in China.

### 2.3. Research Methods

**Figure 1:**
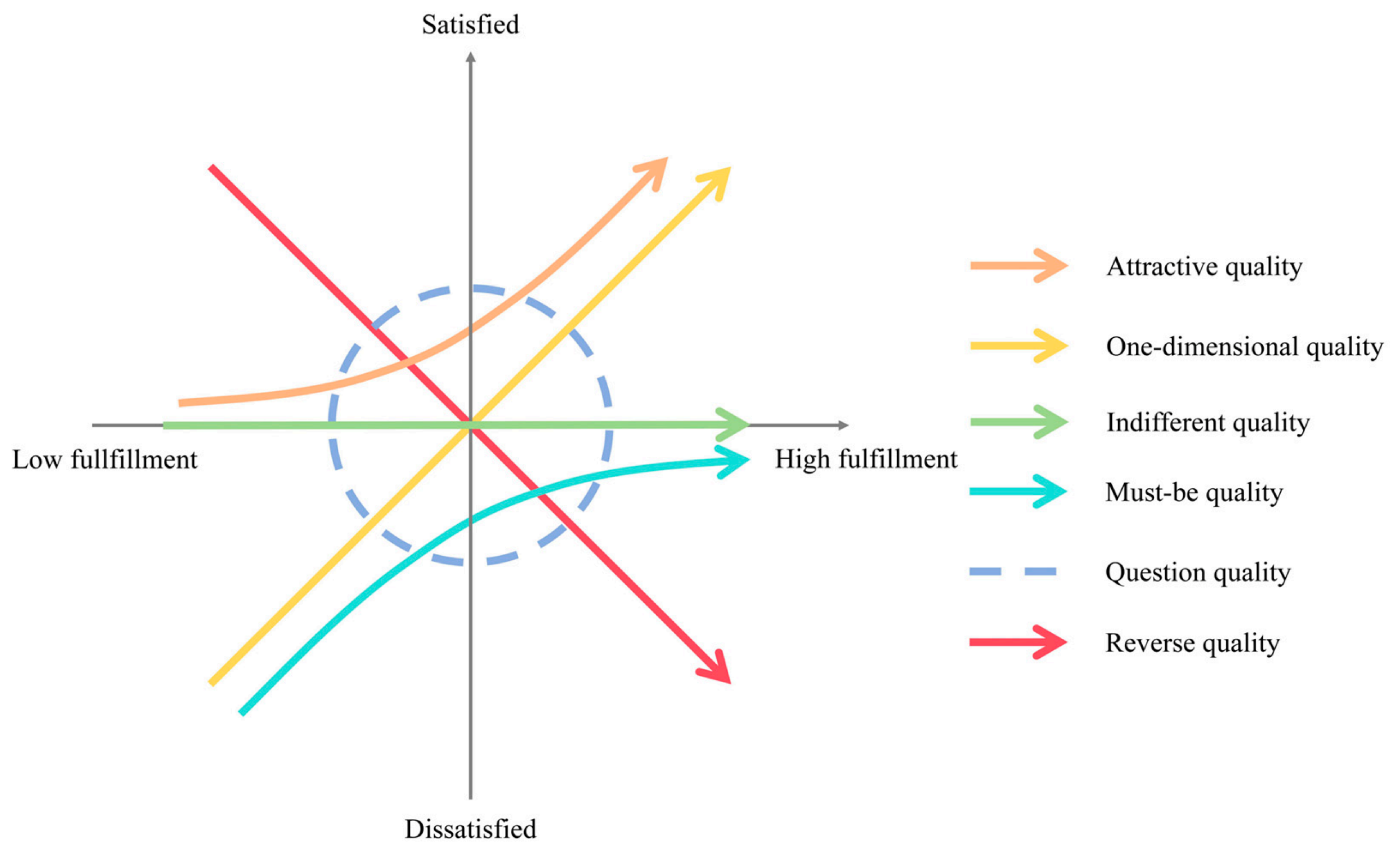
Schematic diagram of the Kano model.

Proposed by Noriaki Kano [17], the Kano model categorizes requirements into five types based on how fulfillment impacts user satisfaction (Figure 1). First, attractive quality (A) refers to features that exceed expectations; their absence does not decrease satisfaction, but their fulfillment significantly enhances it. Second, one-dimensional quality (O) exhibits a positive linear correlation with satisfaction. Third, indifferent quality (I) means fulfillment or absence does not significantly impact satisfaction. Fourth, must-be quality (M) represents fundamental expectations; fulfillment does not greatly increase satisfaction, but absence causes a drastic decline. Finally, reverse quality (R) refers to undesired features that cause dissatisfaction when provided. Additionally, questionable quality (Q) indicates contradictory responses from the participants. However, the Kano model cannot quantitatively determine the relative importance among various requirements, nor can it perform hierarchical and systematic analyses. Since the impact of requirement fulfillment on user satisfaction is influenced by the user’s perceived importance of that specific requirement, Berger [24] recommended evaluating requirement importance simultaneously when utilizing the Kano model to analyze requirement attributes. Therefore, this study further incorporated AHP to analyze the relative importance of each requirement item. Proposed by T.L. Saaty [25] in the 1970s, AHP calculates the relative importance weight of each requirement item, with the sum of overall importance totaling 100%. While the Kano model is straightforward to implement and qualitatively assesses how the fulfillment degree of each requirement affects user satisfaction, the advantage of AHP lies in its capability to hierarchically deconstruct and quantify multi-level requirements into comparable importance weight values. Consequently, the Kano model and AHP complement each other, jointly establishing a comprehensive requirement analysis framework.

### 2.4. Survey Tools

The questionnaire used in this study was jointly developed through a literature review, qualitative interviews, and Delphi expert consultations. First, a systematic search of eight databases—including the Cochrane Library, Embase, PubMed, and Web of Science—was conducted to retrieve literature related to robot-assisted excrement cleaning for inpatients, aiming to understand the current research landscape in this field. Subsequently, semi-structured interviews were conducted with 18 ICU nurses to explore their actual needs regarding the intelligent excreta nursing robot. The interview data were analyzed using thematic analysis, yielding 4 themes and 16 sub-themes. Based on the literature review and qualitative research findings, an initial requirement item pool for an intelligent excreta nursing robot for critically ill patients was constructed. Finally, experts in critical care nursing, nursing management, and nursing robotics were invited to participate in two rounds of Delphi consultation. The response rate for both rounds was 100%. Based on 42 suggestions from the first round and 12 suggestions from the second round, the items were revised to form the final requirement inventory, which consists of 4 primary requirement items and 16 secondary requirement items (Table 1). Following the Kano model framework, the 16 s-level requirement items were transformed into a structured questionnaire. This questionnaire assessed nurses’ actual feelings regarding the presence or absence of a specific requirement feature by having them select from five options (Satisfied, Expected, Indifferent, Tolerable, or Dissatisfied) [26]. This process formulated the Kano model-based requirement attribute analysis questionnaire. Furthermore, an AHP-based requirement importance analysis questionnaire was developed to evaluate the relative importance of each item. This questionnaire utilized pairwise comparisons of requirement items as the question stems, with response options based on the 1 to 9 importance scale proposed by Thomas L. Saaty [27]. A general demographic questionnaire was also included to collect data on gender, age, educational background, professional title, and years of ICU work experience. Prior to the formal survey, a pilot study was conducted. Following the inclusion and exclusion criteria, 20 nurses were recruited to complete the Kano model-based questionnaire, and 2 experienced nursing experts were selected to complete the AHP-based questionnaire. All participants reported that the questionnaires were clearly structured, easy to understand, required no further modifications, and took a reasonable amount of time to complete. The pilot study revealed that the Cronbach’s α coefficient for the Kano model-based questionnaire was 0.92, with 0.91 for the positive questions and 0.99 for the negative questions. A Cronbach’s α coefficient greater than 0.8 indicates excellent reliability; thus, the pilot results confirmed the questionnaire’s suitability for the subsequent formal survey.

### 2.5. Survey Methodology

Within the established regional strata and hospital scope of the multi-stage sampling framework, the research team recruited nurses who met the inclusion and exclusion criteria from each sampled hospital by directly contacting the head nurses and utilizing national academic exchange platforms for nursing professionals. These contact methods served strictly as operational pathways to reach the target hospitals within the predefined sampling framework; the regional stratification structure and the hospital sampling scope were predetermined according to the multi-stage sampling design and remained unaffected by the recruitment channels. The questionnaires were administered anonymously and distributed via the Wenjuanxing (an online survey platform https://www.wjx.cn/) and WeChat (https://weixin.qq.com/) platforms. Upon collection, all retrieved questionnaires were systematically audited, and those containing obvious logical contradictions or systematic errors were excluded.

### 2.6. Statistical Methods

Data entry and statistical analysis were performed using Microsoft Excel 2021 and SPSS (version 27.0). Descriptive statistics were applied to summarize general data, with categorical variables expressed as frequencies and percentages. Continuous variables adhering to a normal distribution were presented as mean ± standard deviation, whereas non-normally distributed data were described using medians and interquartile ranges. Based on the Kano questionnaire results, demand attributes were determined using the classification evaluation reference table [28] (Table 2). The Better-Worse coefficients—specifically, satisfaction influence (SI) and dissatisfaction influence (DSI)—were calculated to classify demand attributes based on percentage distributions. The SI value was calculated as

***SI = (A + O)/(A + O + M + I)*,** (1)

where a value closer to 1 indicates that fulfilling the demand significantly enhances user satisfaction. The DSI value was calculated as

***DSI = −(M + O)/(A + O + M + I)*,** (2)

where a value closer to −1 indicates that failing to meet the demand severely diminishes user satisfaction [24]. An SI-DSI quadrant chart was constructed to categorize demand attributes, using SI as the vertical axis and the absolute value of DSI (|DSI|) as the horizontal axis. The coordinate system was partitioned using the intersections of the mean values of SI and |DSI|. Accordingly, demand items located in the first, second, third, and fourth quadrants were categorized as One-dimensional, Attractive, Indifferent, and Must-be attributes, respectively [29]. Sensitivity was evaluated using the formula

(3) $$R=\sqrt{{SI}^{2}+{DSI}^{2}}-0.707,$$

where a larger R value indicates greater sensitivity in nursing experience [30]. For the AHP questionnaire data, the geometric mean method was employed to aggregate the importance judgments from individual experts [31]. The weights of the judgment matrix were computed using the sum-product method. Global importance weights were derived via hierarchical synthesis, wherein local weights were sequentially multiplied by the weights of their corresponding higher-level demand items [32]. To ensure logical consistency, the consistency check was performed using the consistency ratio formula:

***CR = (λmax – n)/[(n –* **1*) × RI]***,** (4)

A CR indicated acceptable consistency for the matrix [33]. According to the general theory of the Kano model, the priority for function or service provision typically follows the sequence of M > O > A > I [34]. Consequently, an adjustment coefficient (K) was assigned to each demand attribute: K = 4 for M, K = 3 for O, K = 2 for A, and K = 1 for I. Finally, the adjusted demand weights were normalized to determine the ultimate weights of demand importance.

## 3. Results

### 3.1. General Characteristics of the Participants

For the Kano questionnaire, a total of 401 responses were collected. Nineteen questionnaires were manually excluded due to uniform option selections across all items, yielding 382 valid responses and an effective recovery rate of 95.26%. For the AHP questionnaire, 102 responses were retrieved, from which five were manually removed for identical reasons (straight-lining responses). This resulted in 97 valid questionnaires, representing an effective recovery rate of 95.1%. The detailed distribution of valid response rates across different regions is presented in Table 3. The demographic characteristics of the participants are summarized in Table 4.

### 3.2. Reliability and Validity of Questionnaires

The formal survey demonstrated that the Cronbach’s α coefficient for the Kano model-based questionnaire was 0.92 (0.98 for the positive questions and 0.98 for the negative questions). For the mandatory section of the AHP-based requirement importance questionnaire, the Cronbach’s α coefficient was 0.93. All coefficients exceeded 0.8, demonstrating that both questionnaires possessed good reliability. Regarding validity, the ***KMO*** measure of sampling adequacy and Bartlett’s test of sphericity were utilized. For the Kano model-based questionnaire, the overall ***KMO*** value was 0.96 (*p* < 0.05), with 0.96 for the positive questions (*p* < 0.05) and 0.97 for the negative questions (*p* < 0.05). For the mandatory section of the AHP-based questionnaire, the ***KMO*** value was 0.82 (*p* < 0.05). All ***KMO*** values were greater than 0.70 [35], and Bartlett’s test of sphericity reached statistical significance in all cases (*p* < 0.05), indicating that both questionnaires had satisfactory construct validity and well-designed items.

### 3.3. Analysis of Demand Attributes for Intelligent Excreta Nursing Robots Based on the Kano Model

#### 3.3.1. Full-Sample Demand Attribute Analysis

**Figure 2:**
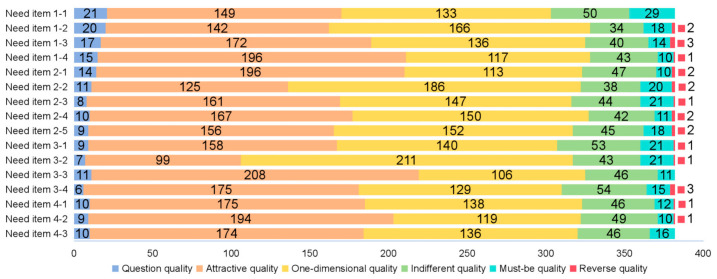
Frequency distribution of requirement attributes.

**Figure 3:**
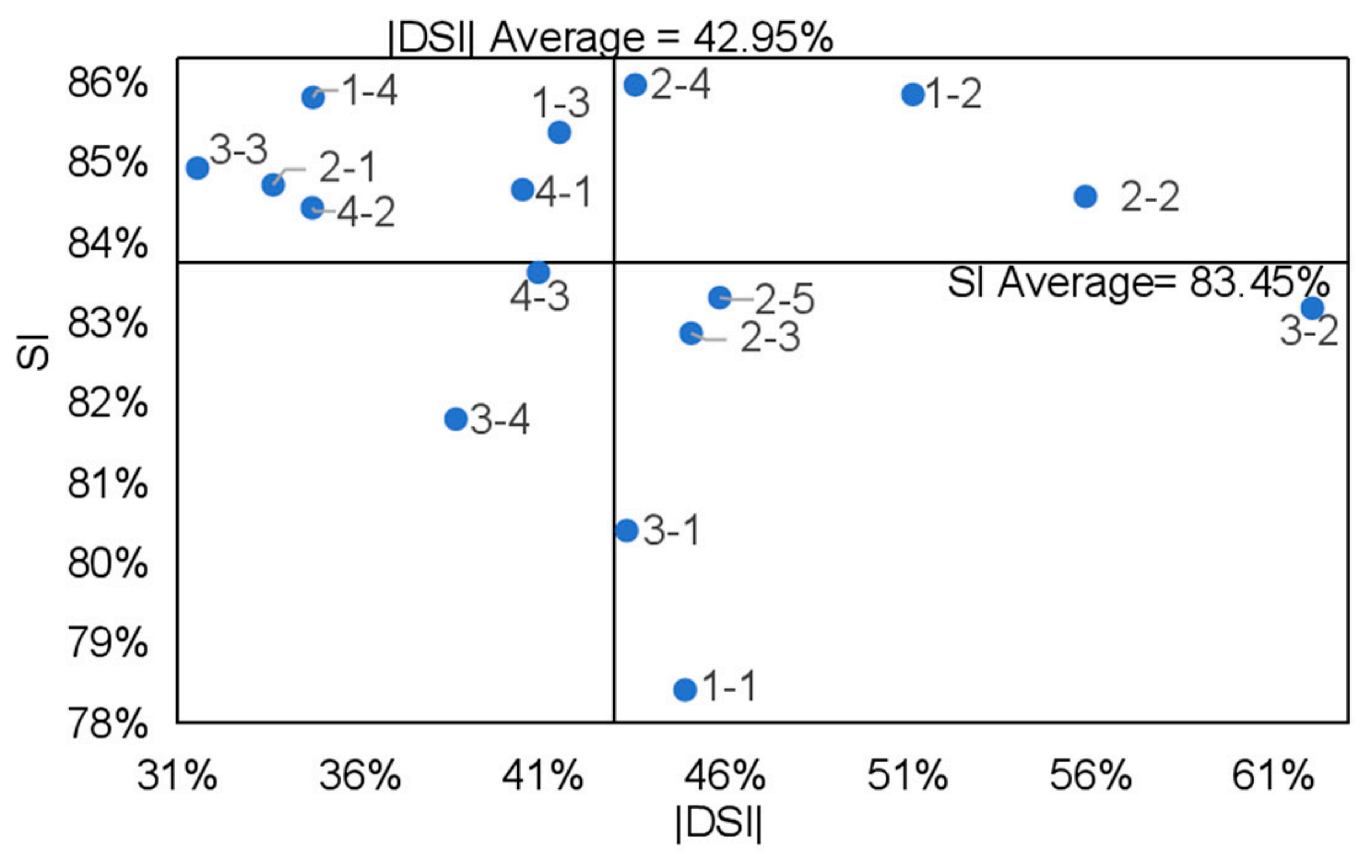
SI-DSI diagram. Note: 1-1: Precise excrement sensing; 1-2: Efficient cleansing of urine and feces; 1-3: Rapid skin drying; 1-4: Post-cleansing skin protection; 2-1: Compatibility with other operations; 2-2: Minimization of use-related injury risks; 2-3: Anomaly prevention and emergency response; 2-4: Status monitoring and notification; 2-5: Comprehensive privacy protection; 3-1: Durable and robust design; 3-2: Comprehensive after-sales support; 3-3: Future functionality expansion; 3-4: Practical affordability and accessibility; 4-1: Friendly human–robot interaction; 4-2: Multi-Device Interconnection System; 4-3: ICU environmental compatibility; SI: satisfaction influence; DSI: dissatisfaction influence.

The frequency statistics for the demand attributes across the 16 s-level demand items are illustrated in Figure 2. Following the exclusion of the R and Q categories from the frequency statistics, the remaining four demand attributes—namely A, O, I, and M—were fully normalized to 100% to calculate the SI, DSI, and sensitivity values, as presented in Table 5. A Kano SI-DSI matrix was subsequently constructed based on these calculated values, as depicted in Figure 3. The demand attribute analysis derived from the SI-DSI matrix reveals the following classifications: the demand items located within the fourth quadrant, including “Precise excrement sensing,” “Anomaly prevention and emergency response,” “comprehensive privacy protection,” “durable and robust design,” and “comprehensive after-sales support,” are identified as must-be quality; those positioned within the first quadrant, specifically “efficient cleansing of urine and feces,” “minimization of use-related injury risks,” and “Status monitoring and notification,” are classified as one-dimensional quality; the items situated in the second quadrant, encompassing “rapid skin drying,” “Post-cleansing Skin Protection,” “Compatibility with other operations,” “future functionality expansion,” “friendly human–robot interaction,” and “Multi-Device Interconnection System,” are categorized as attractive quality; and finally, the items clustered in the third quadrant, namely “practical affordability and accessibility” and “ICU environmental compatibility,” are designated as indifferent quality.

#### 3.3.2. Subgroup Analysis of Demand Attributes

**Figure 4:**
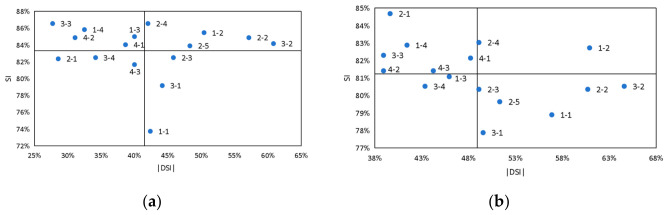

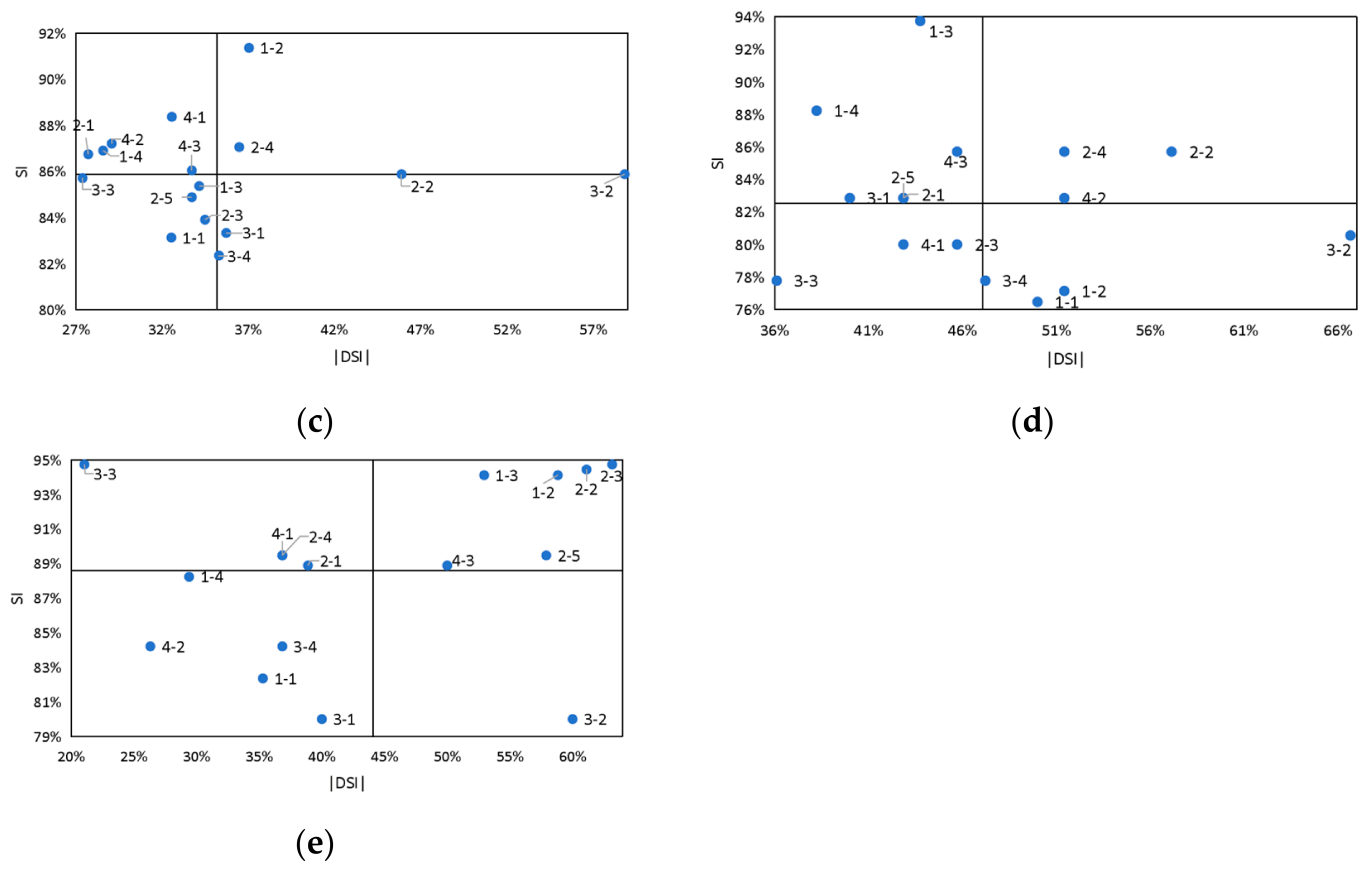
SI-DSI Quadrant Plot Across ICU Nurses with Different Work Experience. (**a**) 1–4 years; (**b**) 5–9 years; (**c**) 10–14 years; (**d**) 15–19 years; (**e**) 20 years or more.

**Figure 5:**
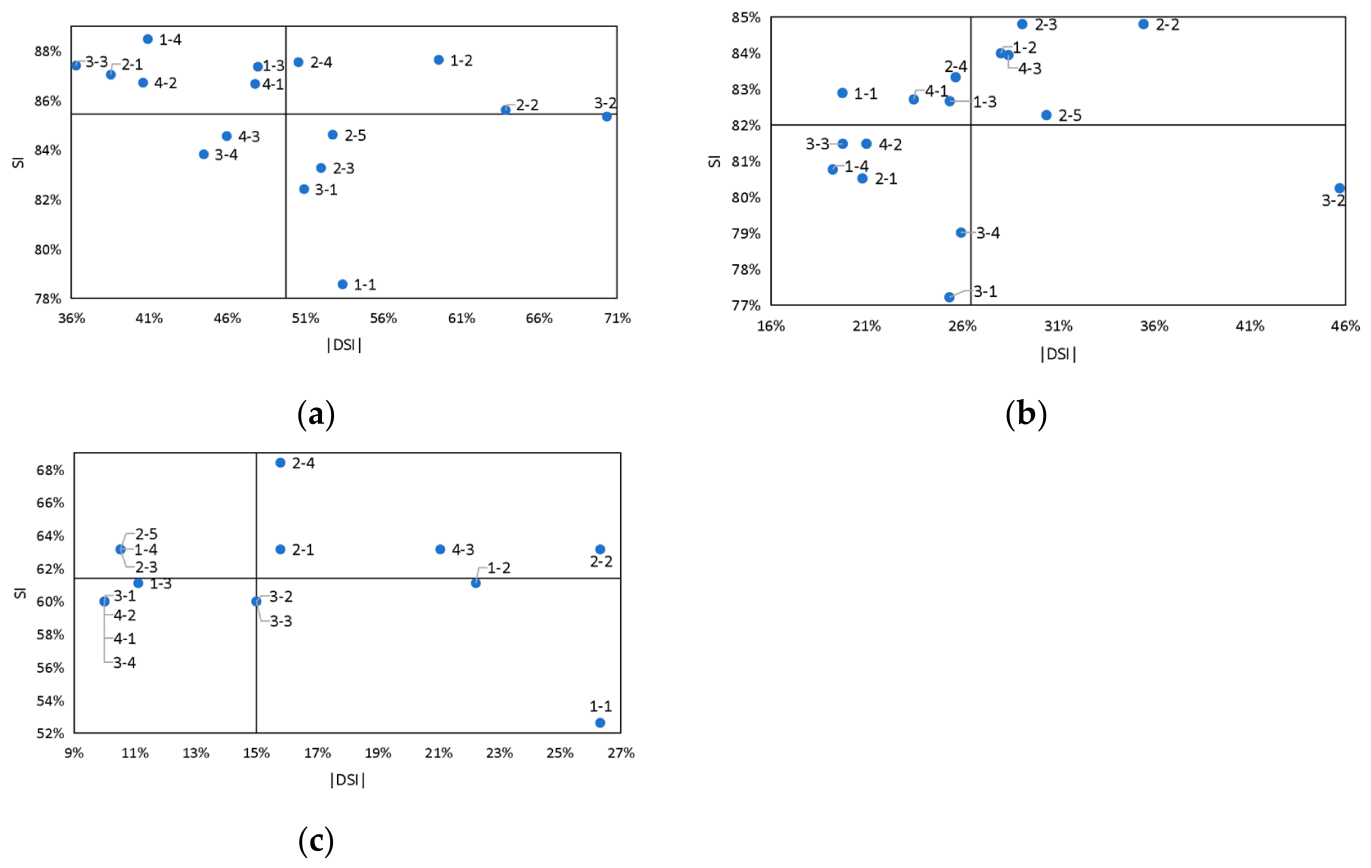
SI-DSI Quadrant Plots Across Different Regions. (**a**) Eastern; (**b**) Central; (**c**) Western.

**Figure 6:**
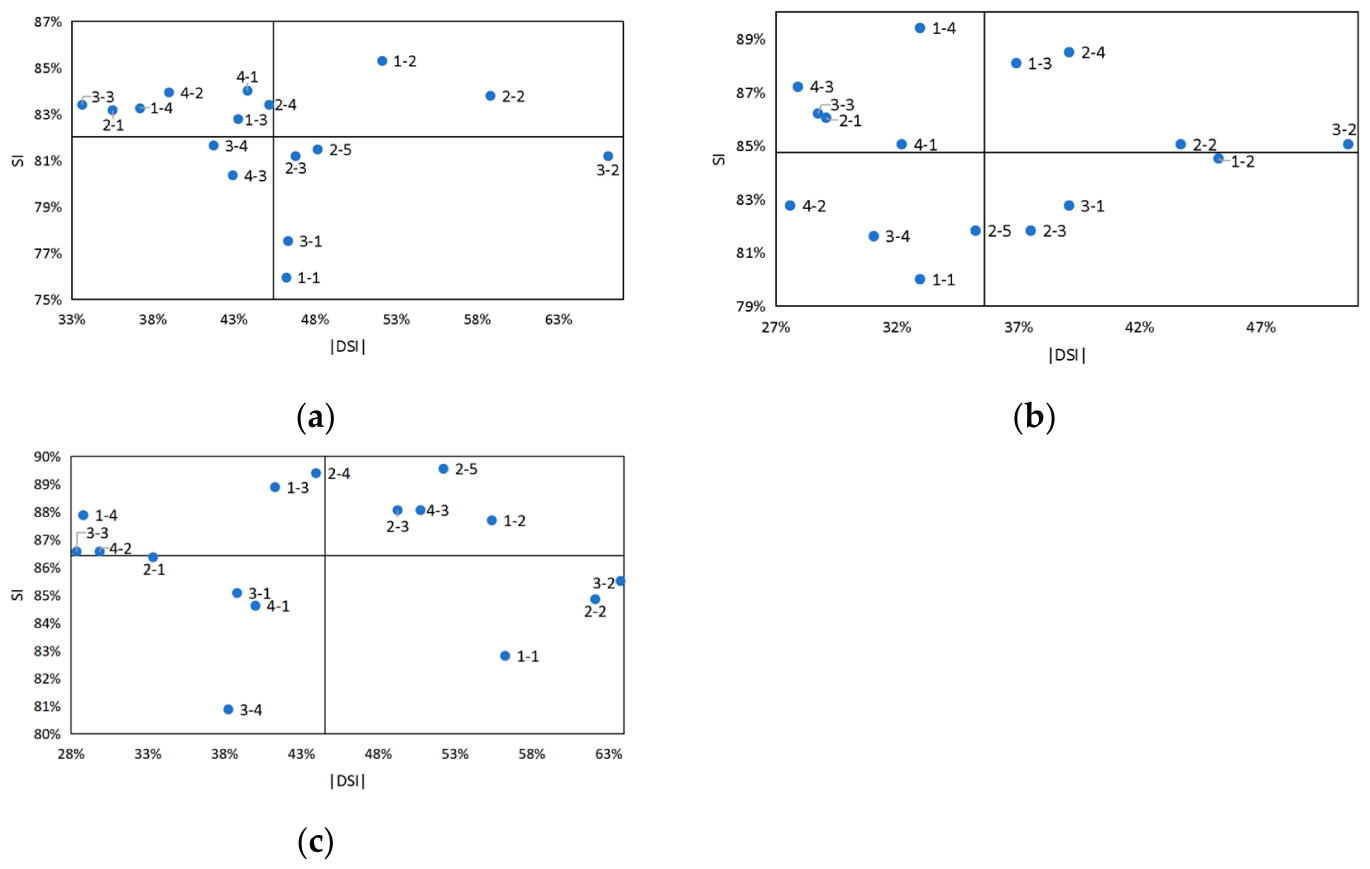
SI-DSI Quadrant Chart across Different Nursing Positions. (**a**) Nurses; (**b**) Nursing team leaders; (**c**) Ward head nurses and above.

The study subjects were divided into five groups based on their years of working experience in the ICU, and the corresponding SI-DSI quadrant chart was plotted (Figure 4). When categorized by region into three groups, the SI-DSI quadrant chart is illustrated in Figure 5. Additionally, the study subjects were stratified into three groups according to their job positions, with the resulting SI-DSI quadrant chart presented in Figure 6. Based on these SI-DSI charts, the demand attribute analysis results for the different subgroups are summarized in Table 6.

### 3.4. Analysis of Requirement Importance for Intelligent Excreta Nursing Robots Based on AHP

#### 3.4.1. Full-Sample Analysis of Requirement Importance

**Figure 7:**
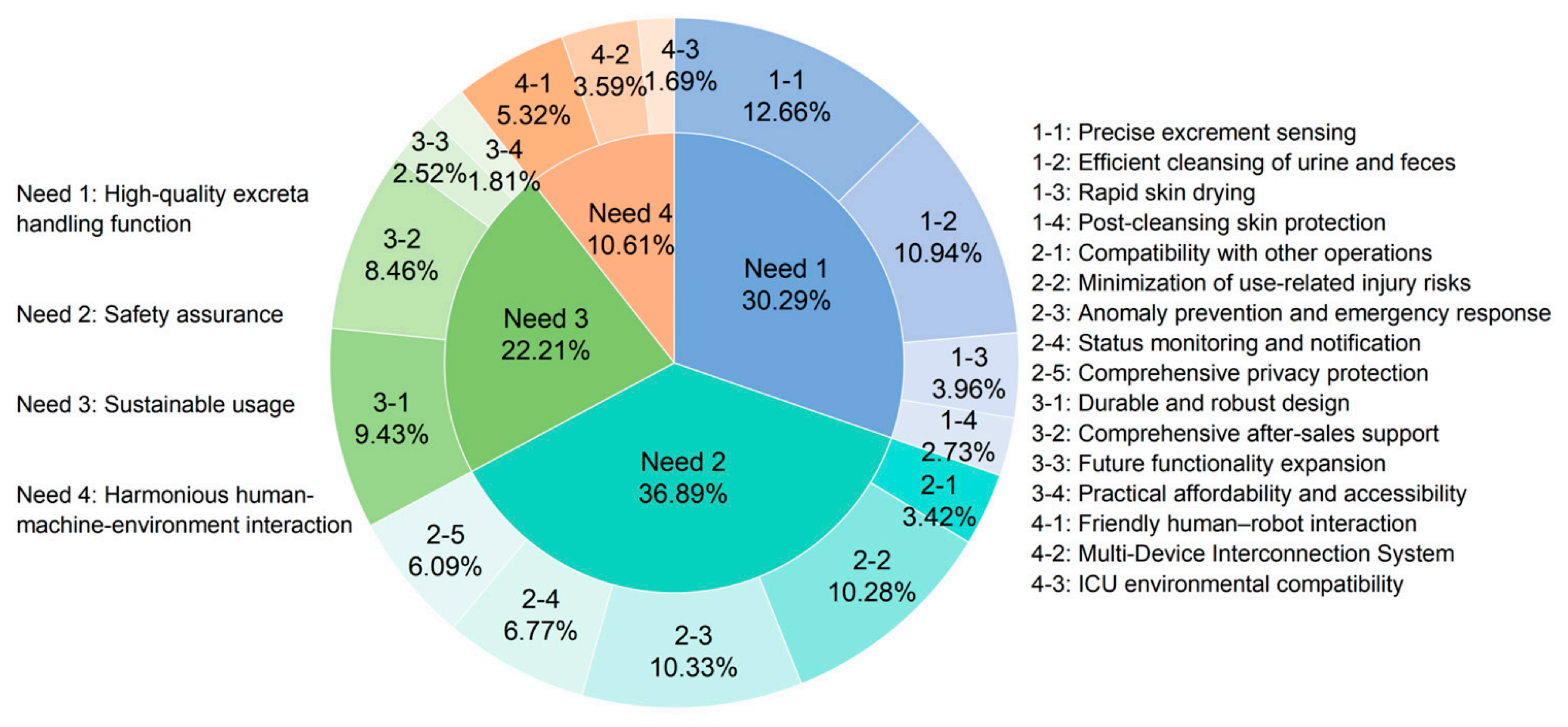
Weight distribution of demand item importance.

The λmax of the judgment matrix for the primary requirement items calculated by AHP was 4.01. According to the RI table, the RI value for a 4-order matrix is 0.89 [36], which yielded a CR of 0.00. The CR values for the remaining four secondary requirement judgment matrices were 0.01, 0.01, 0.00, and 0.00, respectively. All CR values were strictly below 0.1, indicating that all matrices successfully passed the consistency check, thereby validating the weights of requirement importance. Furthermore, the importance weights of each requirement item were adjusted based on the requirement attributes derived from the Kano model to obtain the final importance weights. The results are summarized in Table 7 and Figure 7.

#### 3.4.2. Subgroup Analysis of Demand Importance

**Figure 8:**
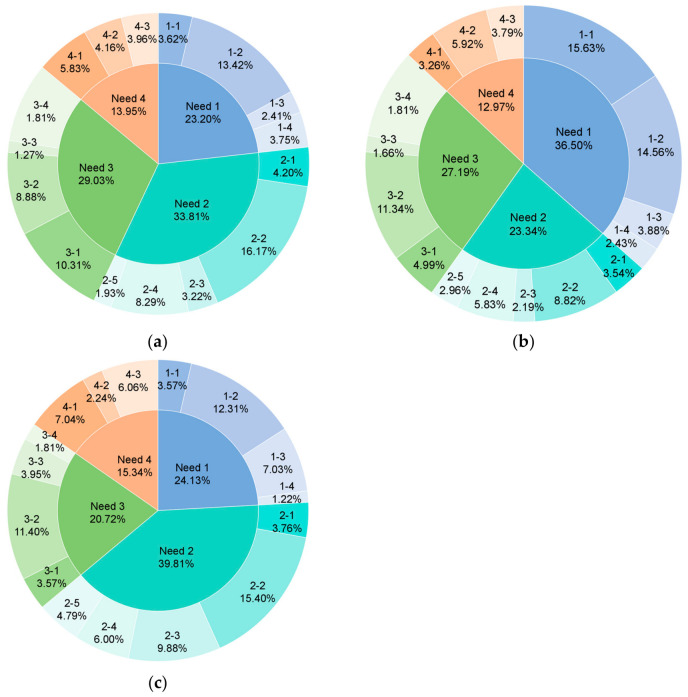
Importance Weights of Demand Items by ICU Work Experience. (**a**) 10–14 years; (**b**) 15–19 years; (**c**) 20 years or more.

**Figure 9:**
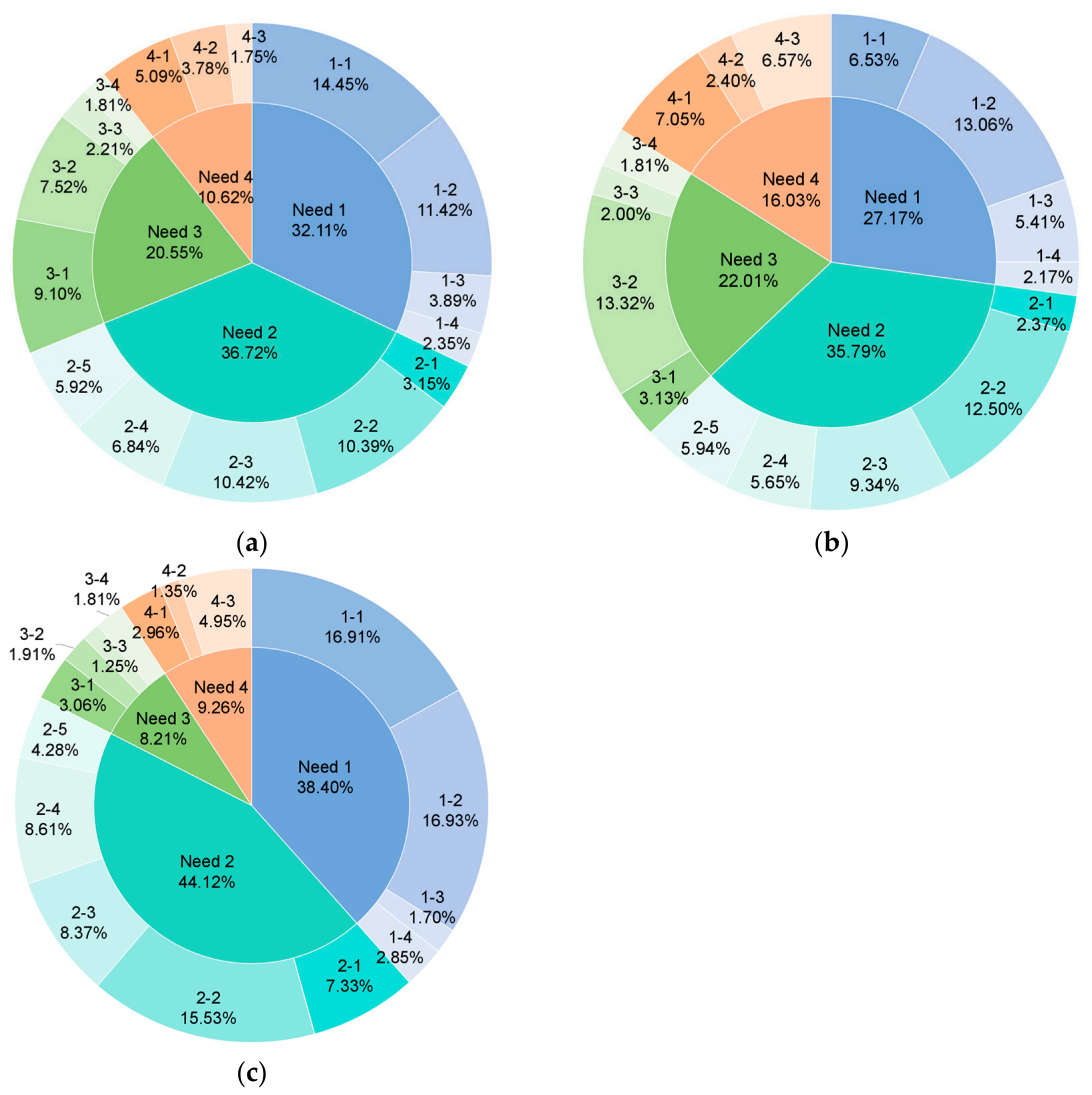
Importance Weights of Demand Items by Region. (**a**) Eastern; (**b**) Central; (**c**) Western.

**Figure 10:**
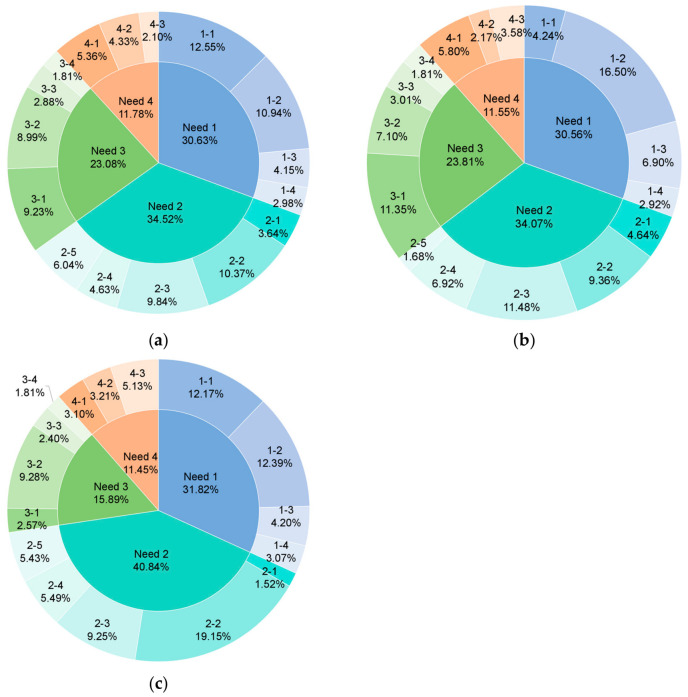
Importance Weights of Demand Items by Position. (**a**) Nurses; (**b**) Nursing team leaders; (**c**) Ward head nurses and above.

Based on their ICU work experience, the participants were divided into three groups, and the importance weights of each item were calculated (Figure 8). Similarly, the participants were categorized into three groups according to region, with the corresponding importance weights of each item presented in Figure 9. Furthermore, the participants were classified into three groups based on their job titles, and the resulting importance weights of each item are shown in Figure 10. All matrices successfully passed the consistency test, confirming the validity of the importance weight values for all items.

## 4. Discussion

Based on the Attractive Quality Theory, this study integrated the Kano model and AHP to determine the attribute classifications and importance weights of various demand items for an intelligent excreta nursing robot among ICU nurses. Specifically, the Kano model elucidated four distinct impact pathways through which these demand items influence user satisfaction. Simultaneously, the AHP was utilized to calculate the baseline importance of each demand item when the cumulative overall importance was set at 100%, after which these weights were further refined based on their respective demand attributes. This integrated analysis combines the intuitive nature of qualitative classification with the precision of quantitative weighting, thereby ensuring that the findings are both scientifically rigorous and highly actionable. Ultimately, this approach provides a robust, demand-driven prioritization framework to guide the design and development of the robot.

### 4.1. The Core Clinical Concern Focuses on Safety Assurance and Superior Functionality

The results of this study indicate that ICU nurses generally perceive safety assurance and superior functionality as their core requirements. This alignment is consistent with the established consensus in prior literature, which posits that nurses expect robots to safely and effectively alleviate the burden of physically intensive tasks [37,38]. Furthermore, quantitative analysis using the AHP weights revealed that ICU nurses prioritize safety assurance over superior functionality. This finding suggests that within the ICU context, the primary concern of nurses is not the technological sophistication of the robot in replacing specific nursing tasks, but rather its capacity to provide secure nursing support within complex clinical environments. This outcome echoes contemporary perspectives in medical artificial intelligence and nursing robotics implementation studies; specifically, in high-risk healthcare settings, clinicians typically prioritize the reliability, safety, and risk management capabilities of technical systems over their degree of automation or algorithmic complexity [39]. Consequently, future research and development of intelligent excreta nursing robots in the ICU must establish a design philosophy that privileges safety assurance. The present study also clarified that among all identified requirements for the intelligent excreta nursing robot, the top four priorities are Precise Excrement Sensing, efficient cleansing of urine and feces, Anomaly prevention and emergency response, and minimization of use-related injury risks. Crucially, these four requirements were categorized as either one-dimensional quality or must-be quality. This provides developers with a direct, clinically oriented framework. Specifically, developers should integrate humidity and ammonia concentration computational data to enhance the robot’s sensitivity to new excreta. They should also construct an adaptive excreta cleaning system based on localization and material recognition technologies. Furthermore, integrating bottom suction mechanisms and instant-stop functions is necessary to prevent and manage anomalies such as collisions or tipping. Finally, utilizing disposable waste collection chambers will prevent robot-related adverse events like cross-infection. Implementing these designs will enable the robot to initiate efficient excreta cleaning via Precise Excrement Sensing while effectively alleviating clinical anxieties among nurses regarding robotic utility. A paradoxical finding emerged regarding “Comprehensive privacy protection,” which was classified as a must-be quality but ranked only eighth in importance. This disparity may be attributed to the inherent nature of must-be quality requirements, which are fundamentally perceived as default expectations. Because this requirement is technologically straightforward to implement, nurses assume that the robot inherently satisfies it, leading to a relative undervaluation of its stated importance. Alternatively, given the critical condition of ICU patients, the perceived importance of privacy security may be marginally secondary to direct physiological safety assurances. Although the importance ranking for privacy protection is relatively lower, this does not imply that the requirement is negligible. Robot developers should not rely solely on hierarchical importance rankings for design decisions, nor should they reduce investment in privacy protection designs based on lower rankings; instead, they must recognize that a must-be quality requirement serves as a signal for mandatory preservation. As noted by Adeyemo et al. [40], patient privacy remains a primary concern in robotic application, and participants in the study by Kang A et al. [41] similarly expressed anxieties regarding privacy breaches from nursing robots. A lack of comprehensive privacy protection in these devices will significantly diminish nurses’ satisfaction with robotic adoption. With the advancement of smart healthcare and interconnected data systems, future nursing robots will increasingly involve patient information acquisition and data transmission. Therefore, robotic design regarding safety assurance must encompass both physical safety and patient data privacy. This necessitates the implementation of data access control, information encryption, and physical privacy shielding to mitigate the risk of patient privacy leaks.

### 4.2. Real-User Feedback Promotes Sustainable Use and Intensified Harmonious Interaction

This study revealed that certain nurse requirements regarding the sustainable usage and interaction of robots merit special attention. This finding slightly contrasts with many engineering-oriented studies that predominantly focus on the specific technical performance of the robots themselves [42,43,44], while paying less attention to the extended after-sales support and human–robot interaction [45]. This discrepancy primarily stems from the fact that this study adopts the perspective of ICU nurses as the end-users to understand their genuine demands. Consequently, it places greater emphasis on the long-term user experience of robots within real nursing environments, rather than focusing solely on the technological advancement from the developers’ perspective [6]. Our findings indicated that nurses perceive “Comprehensive after-sales support” as a must-be requirement, implying that its absence would significantly diminish user satisfaction; notably, it ranked sixth in terms of importance. This result demonstrates that achieving the sustainable usage of robots requires not only a conventional focus on a “Durable and robust design” but also dedicated attention to after-sales support. Therefore, future development of intelligent excreta nursing robots should not be confined to the device hardware design itself. Instead, it is imperative to synchronously establish a well-rounded training system, remote support mechanisms, and rapid maintenance systems to guarantee the sustainable usage of robots in clinical practice. Furthermore, “Friendly human–robot interaction” was identified as an attractive quality, meaning that fulfilling this requirement would remarkably enhance satisfaction. This attribute carried the highest weight of importance among all interaction-related demands, underscoring that a superior human–robot interaction experience plays a pivotal role in the effectiveness of robot engagement. Wong KLY et al. [46] mentioned in their study that healthcare workers harbor concerns regarding the potential increase in workload brought by robots, such as cleaning and charging the equipment. Similarly, Dino MJS et al. [47] posited that human–robot interaction plays a critical role in ensuring the success and efficacy of robots tailored for end-users. Georgadarellis et al. [11] also expressed the need for user-friendly devices that do not introduce additional cognitive or physical burdens. Collectively, these insights suggest that nurses must feel that interacting with robots is effortless and does not impose extra task loads. Hence, robots must be equipped with comprehensive after-sales support, with a parallel focus on the interaction experiences of both nurses and patients. It is worth reflecting on “practical affordability and accessibility” and “ICU environmental compatibility,” both of which were classified as indifferent qualities in our results. The former received a low perceived importance, potentially because the nurses responded in their capacity as end-users rather than purchasers. However, cost remains a critical barrier to the widespread adoption of robots in primary hospitals; thus, robot developers should not overlook economically accessible designs based on the “indifferent” responses of nurses. Regarding the latter, although it ranked last, issues such as device volume and noise have been proven in practical clinical implementations to be realistic impediments to robot promotion. Therefore, environmental compatibility must still be incorporated into early-stage design considerations.

### 4.3. The Clinical Promotion of Intelligent Excreta Nursing Robots in the ICU Is Influenced by Multifaceted Factors

The results of this study indicated that the requirement item “Precise Excrement Sensing” was classified as a must-be quality and ranked first in importance. Meanwhile, “Friendly human–robot interaction” was categorized as an attractive quality, ranking ninth in importance. The item “practical affordability and accessibility” accounted for 1.81% of the total importance weight, and “Comprehensive after-sales support” was identified as a must-be quality (M), ranking sixth. These findings suggest that the successful clinical promotion of intelligent excreta nursing robots in the ICU—which facilitates the transition of excreta care from a manual modality to a more intelligent one—is not merely a technological issue. Rather, it is inextricably linked to staffing, cultural factors, nurse–patient relationships, cost, and training. This multi-dimensional dependence can be attributed to several critical factors. First, if the core technology of the robot is inadequate—for instance, if the automatic excrement sensing technology lacks sensitivity—it could prolong the duration of excrement contact with the patient’s skin, thereby leading to adverse patient outcomes such as incontinence-associated dermatitis. Second, from a staffing perspective, nursing human resources are already in severe shortage. If the nursing labor required for donning, doffing, and terminal processing of the robot outweighs the manpower saved by automated cleaning and disposal, the nurses’ willingness to adopt the technology will be substantially compromised. Third, regarding culture and nurse–patient relationships, having a robot cleanse the patient’s private areas can alleviate the discomfort experienced by patients during manual handling by nurses, which in turn helps maintain a harmonious nurse–patient relationship. Furthermore, in terms of cost, an exorbitant price tag for the robot would aggravate the financial burden on patients, consequently dampening their willingness to utilize it. Conversely, if the robot is backed by a mature and highly efficient training program, it will enable nurses to rapidly master its correct operational protocols. Synthesizing the aforementioned factors, it is highly recommended that hospitals formulate a phased implementation strategy [15]. In the initial introduction phase, hospitals should prioritize selecting robots that satisfy the must-be qualities and one-dimensional qualities that ranked higher in importance. Implementation should commence in ICU beds with relatively stable patients to systematically collect nurse feedback and streamline the nursing workflow. Iterative optimizations can then be executed based on this feedback to minimize implementation resistance before gradually scaling up to the entire ward. Regarding cost accessibility, financial barriers could be mitigated through centralized hospital procurement or leasing models, thereby lowering the economic threshold for adoption.

### 4.4. Requirement Prioritization Guides the Development and Clinical Translation of Intelligent Excreta Nursing Robots

According to the Kano model’s considerations for user satisfaction, the priority of requirement fulfillment generally follows the sequence of must-be quality > one-dimensional quality > attractive quality > indifferent quality [48]. Concurrently, based on the Analytic Hierarchy Process (AHP) considerations for importance, priority must be given to requirements with higher importance weight rankings. Synthesizing both the satisfaction and importance attributes of these requirement items, the future development pathway for intelligent excreta nursing robots should adhere to the following principles. First and foremost, grounded in the principle of “safety assurance,” items such as “Minimization of use-related injury risks,” “Anomaly prevention and emergency response,” and “Comprehensive privacy protection” must be treated as absolute pre-requisites for robot design. Within the complex ICU care environment, robots must not only handle excreta but also incorporate infection control, automatic suspension during anomalies, and patient skin protection to guarantee clinical safety. Second, developers should refine the core technologies within “superior excreta disposal functionality,” specifically focusing on “Precise Excrement Sensing” and “efficient and clean excreta disposal.” Future iterations could integrate multi-source sensing technologies with intelligent algorithms to achieve sensitive recognition of patient excretion status and high cleanliness of waste disposal, thereby enhancing the robot’s usability in authentic clinical settings. Third, to achieve sustainable usage, conventional emphasis on a durable and robust design must be coupled with dedicated after-sales support to improve troubleshooting efficiency and equipment maintenance convenience. Furthermore, regarding robot interaction, emphasis should be placed on the harmony of human–robot interaction. Finally, attention must be paid to the other miscellaneous requirements of nurses for intelligent excreta nursing robots. Referencing the results of this ICU nurse demand analysis during the robot R&D process will help minimize the mismatch between engineering design and actual nursing environments, ultimately improving the clinical adaptability and implementation success rate of these robots. This priority distribution is by no means accidental; rather, it is a direct reflection of the high-risk nature of ICU nursing contexts. Nurses’ acceptance of new technologies is predicated on the prerequisite of “doing no harm”; thus, safety-related requirements naturally occupy the highest cognitive priority, whereas interaction experience requirements recede to a secondary position until absolute safety is guaranteed. This hierarchical logic of “safety first, functionality as the foundation, and experience as the baseline” suggests that in high-risk medical scenarios, the priority structure of user demands is predominantly driven by risk-aversion motivations. These findings offer profound insights not only for robotic engineering design but also hold direct guiding value for clinical implementation and regulatory approval. Specifically, the top-priority requirements identified in this study—”Anomaly prevention and emergency response” and “Minimization of use-related injury risks”—should not merely be viewed as design guidelines. Instead, they should serve as mandatory, rigorous benchmarks for pre-market validation to firmly ensure patient safety.

### 4.5. Generalizability of Findings to Other Healthcare Systems

As this study was conducted within the Chinese healthcare context, applying its conclusions to other healthcare systems requires a distinction between transferable and context-dependent elements. On one hand, the requirement prioritization structure of “safety-first, function-oriented, and experience-supported” stems from the shared risk-aversion logic of users in the high-risk ICU setting, rather than being specific to the Chinese nursing system; therefore, this overall hierarchy possesses high transferability to other healthcare systems. Similarly, the integrated Kano and AHP framework employed in this study is a context-independent, user-centered requirement analysis method that can be directly applied to different countries and institutions. On the other hand, some findings exhibit significant context dependence. For instance, “practical affordability and accessibility” was classified as an indifferent quality, which partly results from Chinese nurses responding as end-users rather than payers, as well as China’s specific medical insurance and centralized procurement models. In healthcare systems with higher out-of-pocket proportions or different reimbursement mechanisms, the perceived importance of financial affordability may increase significantly. Furthermore, the specific weights of various requirements are also influenced by factors such as nurse-to-patient ratios, organizational culture, and cultural attitudes toward privacy and bodily care, which vary considerably across countries. Therefore, it is recommended that other healthcare systems, while adopting the requirement framework and prioritization logic of this study, recalibrate the specific weights in accordance with local nursing staffing, payment and regulatory environments, and cultural backgrounds. These recalibrated weights should undergo localized validation before being utilized to guide research, development, and procurement decisions.

### 4.6. Methodological Implications Derived from the Integration of the Kano Model and AHP

The combined application of the Kano model and AHP in this study establishes an integrated requirement analysis framework, the significance of which is manifested at two distinct levels. First, the two approaches exhibit methodological complementarity. While the Kano model captures requirement attributes grounded in emotional and experiential dimensions, AHP delineates the weight of requirement importance based on cognitive and decision-making dimensions. The resulting requirement analysis framework constructed by fusing these two methodologies effectively bridges the perspective limitations inherent in a single-method approach [49]. Second, this integration exerts a rectifying effect on the interpretation of results. Relying solely on AHP might lead to the oversight of essential requirements that are inherently classified as must-be qualities due to potentially low importance weights. By introducing Kano attributes, this study circumvents the risk of making unilateral decisions based exclusively on AHP weights. These insights suggest that in user requirement research for intelligent medical equipment characterized by high risks and multiple objectives, any single methodology is insufficient to comprehensively map the user’s demand structure. Consequently, the integration of Kano and AHP represents a generalizable and scalable methodology, offering a valuable reference for user-centered requirement research in nursing robots and other smart healthcare devices.

### 4.7. Limitations

Although this study innovatively integrates the Kano model and AHP to systematically analyze the multi-level requirements of ICU nurses—thereby providing user-centered evidence for the research and development of intelligent excreta nursing robots—several inherent limitations must be acknowledged. First, as a cross-sectional study, this research only reflects the requirements of nurses at the specific time of the survey. Consequently, it fails to dynamically observe the evolution of user demands as technology advances and clinical application experience accumulates. Future research should consider longitudinal studies to track these shifting requirements over time. Second, both the Kano model and AHP questionnaires rely heavily on self-reported data from participants, meaning the results may be subject to the respondents’ personal experience, cognitive biases, and variations in institutional environments. Third, this study was conducted within a single healthcare system in China. Despite covering multiple regions and hospitals, variations across countries regarding nursing workforce allocation, payment and regulatory systems, organizational culture, and attitudes toward privacy and physical care may alter the attribute classification and importance weights of various needs. Therefore, although the overall priority logic of the needs demonstrates good cross-system transferability, the specific weights require localized calibration and validation prior to international implementation. Future research could incorporate cross-cultural and multi-country comparative studies to verify these findings. Fourth, this study primarily focused on the perspective of nurses, without directly involving other key stakeholders, such as patients and their families. As a result, our understanding of the clinical implementation ecosystem for intelligent excreta nursing robots remains incomplete; future studies could integrate a multi-stakeholder perspective to perform a comprehensive requirement analysis. Finally, while the integrated Kano–AHP framework was successfully applied for requirement analysis, this study has not yet established a direct link between the requirement analysis and actual clinical implementation outcomes. Future endeavors could incorporate real-world studies, implementation science frameworks, and interventional trials to further validate the tangible impacts of fulfilling high-priority requirements on nursing quality, staff workload, and patient outcomes. Furthermore, although this study predetermined the regional stratification and hospital scope based on a multi-stage sampling framework, the actual participant recruitment process within each hospital relied on forwarding by head nurses and dissemination via nursing academic platforms. Consequently, this approach exhibited certain characteristics of convenience sampling, which might have introduced self-selection bias and affected the representativeness of the sample. Future research could implement intra-hospital random sampling within the established sampling framework to enhance sample representativeness.

Overall, the pivotal significance of this study lies in the integrated utilization of the Kano model and AHP. By simultaneously accounting for the impact of various requirements on user satisfaction and incorporating the users’ subjective perspectives on importance, this dual approach precisely captures nurses’ genuine needs and explicitly establishes the aforementioned priority order for requirement fulfillment. Grounded firmly in the empirical demands of nurses—the core end-users of robotic technology—this priority sequence provides a scientifically rigorous basis for decision-making regarding the clinical adaptation and promotion of intelligent excreta nursing robots. Furthermore, it offers a valuable and generalizable methodological reference for user requirement research involving other intelligent medical devices. Ultimately, by gaining deep insights into and systematically addressing the complex demands of nurses as terminal users, this research effectively bridges the persistent chasm between technological advancement and actual clinical application.

## 5. Conclusions

Based on the Attractive Quality Theory, this study coupled the Kano model with AHP to systematically analyze nurses’ requirements for intelligent excreta nursing robots, establishing a well-defined sequence of requirement priority fulfillment. These findings effectively enhance the targeted and scientific nature of intelligent excreta nursing robot design, providing robust user-centered empirical evidence for functional optimization, R&D decision-making, and clinical promotion. Furthermore, this study offers a valuable reference for the clinical implementation of nursing robots against the backdrop of smart ICU construction. Future research and development of intelligent excreta nursing robots for the ICU should not only focus on upgrading excreta disposal performance but also place greater strategic emphasis on nursing safety and sustainable usability.

## Figures and Tables

**Table 1:** ICU nurses’ requirements for intelligent excreta nursing robots.

Level 1 Item Code	Level 1 Item Name	Level 2 Item Code	Level 2 Item Name	Item Description
1	High-quality excreta handling function	1-1	Precise excrement sensing	Real-time and precise identification of new excreta categories (urine, feces, or a mixture of both); accurately determines the end of excretion to collect waste.
		1-2	Efficient cleansing of urine and feces	Defined application targets and adjustable cleaning parameters; capable of rapid warm-water cleaning without leakage, reducing manual labor.
		1-3	Rapid skin drying	Following the hydro-cleansing process, temperature-adjustable airflow is utilized to rapidly dry the patient’s skin, ensuring complete dryness even within skin folds.
		1-4	Post-cleansing skin protection	Following skin drying, the system automatically identifies high-risk areas to apply emollients or skin barrier agents, providing a visual confirmation of the application effect to prevent incontinence-associated dermatitis.
2	Safety assurance	2-1	Compatibility with other operations	Compatible with catheters, wounds, special and routine body positions, monitoring probes, routine and shift-change skin observation, and collection of excreta specimens; capable of being removed with one key during emergency rescue.
		2-2	Minimization of use-related injury risks	The clinical application of the robot must strictly adhere to operational protocols to minimize adverse events, such as catheter-induced injury, electric leakage, allergies, and skin damage, with a specific focus on preventing infection and cross-infection.
		2-3	Anomaly prevention and emergency response	Equipped with mechanisms for preventing and responding to anomalies, such as operational permission management, alarm sounds and information transmission, built-in backup power supply, high-pressure dredging of blockages, and emergency pause for patient abnormalities.
		2-4	Status monitoring and notification	Precisely and intuitively recording and analyzing the patient’s excretion (color, consistency, volume, timing, etc.), skin status (integrity, color, dermatitis, etc.), and robot status (consumables, self-check, etc.); providing clear prompts if there is an abnormality, with a viewing window for manual inspection.
		2-5	Comprehensive privacy protection	The robot effectively covers the patient’s private parts and sets access permissions to protect the security of patient data.
3	Sustainable usage	3-1	Durable and robust design	The robot must be designed for a long service life and adopt wear-resistant and impact-resistant structures.
		3-2	Comprehensive after-sales support	Providing a full range of after-sales services including video tutorials, periodic testing, replacement with backup machines, timely after-sales response, and correctable upgrades.
		3-3	Future functionality expansion	In the future, it can be developed towards functional expansion, integrating abdominal hot compresses, massage, excrement testing, perineal care, patient mobilization, and integration with the smart hospital IoT platform.
		3-4	Practical affordability and accessibility	The robot’s procurement models, pricing, consumables, and maintenance costs align with the financial capabilities of various hospital tiers and patients.
4	Harmonious human–machine–environment interaction	4-1	Friendly human–robot interaction	Can customize operations, work silently, and use low brightness at night to keep patients comfortable; features troubleshooting guides, simplified operational interfaces and programs, and minimal manual operations to make interactions convenient for nurses.
		4-2	Multi-device interconnection System	The robot links with electronic medical systems, excretion-related medical equipment, and mobile terminals, enabling smooth and error-free information transmission among them.
		4-3	ICU environmental compatibility	Can withstand disinfection, features plain colors, a modular compact size, integrated storage for long cables, automatic charging, and deodorization, making it harmoniously compatible with the ICU environment.

**Table 2:** Kano model classification evaluation comparison table.

Answers		Do Not Possess (Negative Question)				
		Satisfied	Expected	Indifferent	Tolerable	Dissatisfied
Possess (affirmative question)	Satisfied	Q	A	A	A	O
	Expected	R	I	I	I	M
	Indifferent	R	I	I	I	M
	Tolerable	R	I	I	I	M
	Dissatisfied	R	R	R	R	Q

Note: Q: questionable quality; A: attractive quality; O: one-dimensional quality; R: reverse quality; I: indifferent quality; M: must-be quality.

**Table 3:** Valid response rates by region.

Region Questionnaire	Attributes of Requirements Items Questionnaire	Questionnaire for Importance Analysis of Requirement Items
Eastern	94.24%	92.98%
Central	97.67%	100.00%
Western	100.00%	85.71%

**Table 4:** General Characteristics of the Participants.

Item		Nurses Completing the Need Item Attribute Analysis Questionnaire (*n* = 382)	Experienced Nursing Experts Completing the Questionnaire on Requirement Item Importance Analysis (*n* = 97)
Gender [***n (%)***]	Male	43 (11.26)	16 (16.49)
	Female	339 (88.74)	81 (83.51)
Age [years, ***M (P25, P75)]***		35.00 (30.00, 38.00)	38.00 (35.00, 43.00)
Total years of work experience [years, ***M (P25, P75)***]		12.00 (8.00, 17.00)	15.00 (13.00, 21.00)
Years of ICU work experience [years, ***M (P25, P75)***]		7.00 (3.00, 11.00)	13.00 (10.00, 18.00)
Education Level [***n (%)***]	Junior college	3 (0.79)	0 (0.00)
	Undergraduate	351 (91.88)	84 (86.60)
	Postgraduate	28 (7.33)	13 (13.40)
Technical title [***n (%)***]	Nurse	9 (2.36)	
	Senior nurse	128 (33.51)	
	Nurse-in-charge	182 (47.64)	65 (67.01)
	Associate chief senior nurse	59 (15.45)	28 (28.87)
	Chief senior nurse	4 (1.05)	4 (4.12)
Position [***n (%)***]	Nurse	223 (58.38)	29 (29.90)
	Nursing team leader	88 (23.04)	35 (36.08)
	Ward head nurse	63 (16.49)	26 (26.80)
	Department head nurse	5 (1.31)	5 (5.15)
	Deputy director of nursing department	3 (0.79)	2 (2.06)
Nursing competency level [***n (%)***]	N0	3 (0.79)	
	N1	49 (12.83)	
	N2	138 (36.13)	13 (13.40)
	N3	109 (28.53)	32 (32.99)
	N4	83 (21.73)	52 (53.61)
Research field [***n (%)***]	Excretion care		36 (37.11)
	Critical care		97 (100)
	Intelligent nursing		12 (12.37)
	Nursing Management		30 (30.93)

**Table 5:** Calculation results of demand attribute parameters.

Codes of Items	Names of Items	A (%)	O (%)	I (%)	M (%)	SI (%)	DSI (%)	Needs Attributes	Sensitivity
1-1	Precise excrement sensing	41.27%	36.84%	13.85%	8.03%	78.12%	−44.88%	M	0.19
1-2	Efficient cleansing of urine and feces	39.44%	46.11%	9.44%	5.00%	85.56%	−51.11%	O	0.29
1-3	Rapid skin drying	47.51%	37.57%	11.05%	3.87%	85.08%	−41.44%	A	0.24
1-4	Post-cleansing skin protection	53.55%	31.97%	11.75%	2.73%	85.52%	−34.70%	A	0.22
2-1	Compatibility with other operations	53.55%	30.87%	12.84%	2.73%	84.43%	−33.61%	A	0.20
2-2	Minimization of use-related injury risks	33.88%	50.41%	10.30%	5.42%	84.28%	−55.83%	0	0.30
2-3	Anomaly prevention and emergency response	43.16%	39.41%	11.80%	5.63%	82.57%	−45.04%	M	0.23
2-4	Status monitoring and notification	45.14%	40.54%	11.35%	2.97%	85.68%	−43.51%	O	0.25
2-5	Comprehensive privacy protection	42.05%	40.97%	12.13%	4.85%	83.02%	−45.82%	M	0.24
3-1	Durable and robust design	42.47%	37.63%	14.25%	5.65%	80.11%	−43.28%	M	0.20
3-2	Comprehensive after-sales support	26.47%	56.42%	11.50%	5.61%	82.89%	−62.03%	M	0.33
3-3	Future functionality expansion	56.06%	28.57%	12.40%	2.96%	84.64%	−31.54%	A	0.20
3-4	Practical affordability and accessibility	46.92%	34.58%	14.48%	4.02%	81.50%	−38.61%	I	0.19
4-1	Friendly human–robot interaction	47.17%	37.20%	12.40%	3.23%	84.37%	−40.43%	A	0.23
4-2	Multi-Device Interconnection System	52.15%	31.99%	13.17%	2.69%	84.14%	−34.68%	A	0.20
4-3	ICU environmental compatibility	46.77%	36.56%	12.37%	4.30%	83.33%	−40.86%	I	0.22

Note: A: attractive quality; O: one-dimensional quality; I: indifferent quality; M: must-be quality; SI: satisfaction influence; DSI: dissatisfaction influence.

**Table 6:** Analysis of Need Attributes Among Different Nurse Subgroups.

Grouping Criteria	Groups	Requirement Items															
		1-1	1-2	1-3	1-4	2-1	2-2	2-3	2-4	2-5	3-1	3-2	3-3	3-4	4-1	4-2	4-3
ICU Experience	1–4 years	M	O	A	A	I	O	M	O	O	M	O	A	I	A	A	I
	5–9 years	M	O	I	A	A	M	M	O	M	M	M	A	I	A	A	A
	10–14 years	I	O	I	A	A	M	I	O	I	M	M	I	M	A	A	A
	15–19 years	M	M	A	A	A	O	I	O	A	A	M	I	M	I	O	A
	20 years or more	I	O	O	I	A	O	O	A	O	I	M	A	I	A	I	O
Region	Eastern	M	O	A	A	A	O	M	O	M	M	M	A	I	A	A	I
	Central	A	O	A	I	I	O	O	A	O	I	M	I	I	A	I	O
	Western	M	M	I	A	O	O	A	O	A	I	I	I	I	I	I	O
Position	Nurses	M	O	A	A	A	O	M	A	M	M	M	A	I	A	A	I
	Nursing team leaders	I	M	O	A	A	O	M	O	I	M	O	A	I	A	I	A
	Ward head nurses and above	M	O	A	A	I	M	O	A	O	I	M	A	I	I	A	O

**Table 7:** Weight calculation results for importance of intelligent excreta nursing robot requirements.

Level 1 Item	Importance Degree of Level 1 Item	Ranking of Importance Degrees of Level 1 Item	Level 2 Item	Importance Degree of Level 2 Item	Ranking of Importance Degrees of Level 2 Item	Needs Attributes
High-quality excreta handling function	30.29%	2	Precise excrement sensing	12.66%	1	M
			efficient cleansing of urine and feces	10.94%	2	O
			Rapid skin drying	3.96%	10	A
			Post-cleansing skin protection	2.73%	13	A
Safety assurance	36.89%	1	Compatibility with other operations	3.42%	12	A
			Minimization of use-related injury risks	10.28%	4	0
			Anomaly prevention and emergency response	10.33%	3	M
			Status monitoring and notification	6.77%	7	O
			Comprehensive privacy protection	6.09%	8	M
Sustainable usage	22.21%	3	Durable and robust design	9.43%	5	M
			Comprehensive after-sales support	8.46%	6	M
			Future functionality expansion	2.52%	14	A
			practical affordability and accessibility	1.81%	15	I
Harmonious human–machine–environment interaction	10.61%	4	Friendly human–robot interaction	5.32%	9	A
			Multi-device interconnection system	3.59%	11	A
			ICU environmental compatibility	1.69%	16	I

Note: A: attractive quality; O: one-dimensional quality; I: indifferent quality; M: must-be quality.

## Data Availability

The data presented in this study are available on request from the corresponding author. The data are not publicly available due to privacy restrictions.

## Abbreviations

The following abbreviations are used in this manuscript:

ICU	Intensive care unit
AHP	Analytic hierarchy process
M	Must-be quality
O	One-dimensional quality
A	Attractive quality
I	Indifferent quality
R	Reverse quality
Q	Questionable quality
SI	Satisfaction influence
DSI	Dissatisfaction influence
